# Effects of Green Tea Extract Epigallocatechin-3-Gallate on Oral Diseases: A Narrative Review

**DOI:** 10.3390/pathogens13080634

**Published:** 2024-07-29

**Authors:** Yizhen Li, Lei Cheng, Mingyun Li

**Affiliations:** State Key Laboratory of Oral Diseases, National Clinical Research Center for Oral Diseases, West China School of Stomatology, Sichuan University, Chengdu 610041, China; liyizhen@stu.scu.edu.cn

**Keywords:** epigallocatechin-3-gallate, oral health, dental caries, pulpitis, periodontal diseases, oral mucosal diseases, salivary gland diseases, oral squamous cell carcinoma

## Abstract

Objectives: Oral diseases are among the most prevalent diseases globally. Accumulating new evidence suggests considerable benefits of epigallocatechin-3-gallate (EGCG) for oral health. This review aims to explore the role and application of EGCG in main oral diseases. Methods: This narrative review thoroughly examines and summarizes the most recent literature available in scientific databases (PubMed, Web of Science, Scopus, and Google Scholar) reporting advances in the role and application of EGCG within the dental field. The major keywords used included “EGCG”, “green tea extract”, “oral health”, “caries”, “pulpitis”, “periapical disease”, “periodontal disease”, “oral mucosa”, “salivary gland”, and “oral cancer”. Conclusions: EGCG prevents and manages various oral diseases through its antibacterial, anti-inflammatory, antioxidant, and antitumor properties. Compared to traditional treatments, EGCG generally exhibits lower tissue irritation and positive synergistic effects when combined with other therapies. Novel delivery systems or chemical modifications can significantly enhance EGCG’s bioavailability, prolong its action, and reduce toxicity, which are current hotspots in developing new materials. Clinical significance: this review provides an exhaustive overview of the biological activities of EGCG to major oral diseases, alongside an exploration of applications and limitations, which serves as a reference for preventing and managing oral ailments.

## 1. Introduction

According to processing techniques, tea can be classified into four main types: white tea, green tea, oolong tea, and black tea [1]. In recent years, green tea received increasing attention due to its health potential. The catechins in green tea polyphenols, including catechin (C), gallocatechin (GC), epicatechin (EC), and epigallocatechin (EGC), as well as gallated catechins such as epicatechin gallate (ECG), catechin gallate (CG), gallocatechin gallate (GCG), and epigallocatechin gallate (EGCG), are believed to be responsible for many of its biological properties [2]. Among these, EGCG, the most abundant green tea catechin, accounting for 40 to 48% of the total catechin content, is recognized as the catechin with the strongest biological activity. Each gram of dried tea leaves contains approximately 10–50 mg of EGCG, corresponding to about 10–80 mg of EGCG per cup of green tea (235 mL) [3], depending on the type of tea, the amount of tea used, brewing time, water temperature, and processing methods of the green tea. Matcha, a type of powdered green tea, has a considerably higher polyphenol content than infusions made by steeping tea leaves because catechins have low thermal stability and limited solubility in water. Additionally, matcha is often used as an additive to improve food flavor, significantly increasing catechin intake [4]. EGCG is mainly found in green tea leaves or matcha, with relatively lower levels in white tea and oolong tea, and the lowest levels in fully fermented teas such as black tea. Additionally, catechins are present in raspberries and cocoa, but in much lower amounts than tea [5]. Currently, the primary method for extracting EGCG involves 70% ethanol extraction of catechins followed by separation and purification through chromatography [6,7].

**Figure 1:**
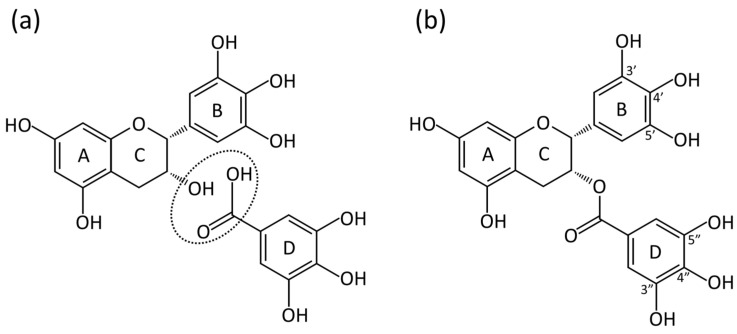
Chemical structure and origin of epigallocatechin gallate. (**a**) Chemical structures of epigallocatechin (EGC, above) and gallic acid (below). Epigallocatechin gallate (EGCG) is formed via the esterification of the circled functional groups of the two reactants. (**b**) Chemical structure of epigallocatechin gallate (EGCG). The two hydroxylated aromatic rings, A and B, are connected by a cyclic pyran ring, C; the aromatic ring D is part of the galloyl moiety.

The chemical structure of EGCG comprises two benzene rings (A and B), a dihydropyran heterocycle ring (C) with two chiral carbon centers at positions 2 and 3, and a gallate moiety (D-ring) as presented in Figure 1. With the vicinal trihydroxy group participating in electron delocalization, the hydroxyl groups in the pyrogallol moiety (B-ring) scavenge superoxide anions, and the galloyl moiety (D-ring) helps in scavenging hydroxyl radicals, enabling each EGCG molecule to scavenge six $O_{2}^{-}$· or ·OH radicals [8]. This activity underpins EGCG’s antioxidant potential against oxidative stress. However, these highly active trihydroxy groups also make EGCG susceptible to oxidation and the generation of reactive oxygen species (ROS) in air, particularly under neutral or alkaline pH conditions [9]. Additionally, EGCG exhibits metal chelation activity via its B- and D-rings [10].

EGCG exhibits various physiological activities, such as anti-infection, anti-inflammation, antioxidation, and anticancer effects [11]. Numerous studies showed that EGCG has inhibitory activity against various pathogenic microorganisms, including many Gram-negative and Gram-positive bacteria, as well as some fungi, viruses, and prions, making it a broad-spectrum antimicrobial agent [12]. It also has significant anti-inflammatory effects by modulating gene expression and molecular signaling pathways, inhibiting the secretion of various inflammatory factors, and reducing the infiltration of inflammatory cells, effectively alleviating tissue inflammation [13]. Additionally, as a green tea polyphenol, EGCG possesses antioxidant activity, which can alleviate cellular oxidative stress by scavenging ROS and regulating intracellular signals [14,15]. In biological studies conducted in vitro and in vivo, EGCG demonstrated anticancer activity against 15 cancers, including breast, lung, liver, gastric, oral, and colorectal cancers [16]. The mechanisms of anticancer activities of EGCG include inhibition of proliferation, adhesion, migration, invasion, metastasis, suppression of angiogenesis, induction of apoptosis, and enhancement of the sensitivity of the immune system to tumors [17].

Oral diseases are among the most prevalent diseases globally and impose serious health and economic burdens, greatly affecting the quality of life of patients [18]. EGCG, as a natural and easily accessible plant extract, has a good application prospect in oral diseases. EGCG improves the oral microbiota and significantly inhibits the activity and virulence factors of pathogenic bacteria closely associated with infectious oral diseases such as *Streptococcus mutans* and *Porphyromonas gingivalis* [19]. EGCG impedes the progression of oral squamous cell carcinoma (OSCC) through mechanisms involving the induction of oxidative stress and apoptosis in cancer cells, as well as the inhibition of tumor invasion [20]. New dental materials relying on the anti-inflammatory properties of EGCG were also widely applied in treatment [21].

Currently, there is a lack of comprehensive reviews summarizing the recent advances in the bioactivity of EGCG against various major oral diseases. Therefore, we review the recent research on the role and application of EGCG in major oral diseases, including dental caries, pulpal and periapical diseases, periodontal diseases, oral mucosal diseases, salivary gland diseases, and oral cancer, aiming to provide a theoretical basis for the development and application of natural drugs.

## 2. Materials and Methods

### 2.1. Search Strategies

A comprehensive search of the literature was conducted using scientific databases (PubMed, Web of Science, Scopus, and Google Scholar). The search was first performed in October 2023, but was updated in July 2024 during peer review, in the event that new references became available since the manuscript was initially prepared.

The keywords used for the search included “EGCG”, “green tea extract”, “oral health”, “caries”, “pulpitis”, “periapical disease”, “periodontal disease”, “oral mucosa”, “salivary gland”, and “oral cancer”. Controlled vocabularies were employed when appropriate, along with synonyms and alternate spellings.

The references of the identified records were imported into Endnote as a Research Information Systems file to remove the duplicates.

### 2.2. Inclusion and Exclusion Criteria

The following studies were included in the literature review in accordance with the following inclusion criteria: (1) studies focused on the properties, applications, and/or advancements in EGCG; (2) studies performed in vitro and/or in vivo; (3) clinical research; and (4) systematic reviews.

The following studies were excluded from the review in accordance with the following exclusion criteria: (1) studies not addressed to the dentistry field; (2) studies published over 10 years, especially highly similar to the newest studies; (3) studies not available in English; and (4) preprints.

Papers published in the last five years were preferentially included.

### 2.3. Data Extraction

The initial search yielded a total of 2361 records. After removing duplicates, 1496 records remained. Titles and abstracts were screened by three independent reviewers to identify studies meeting the inclusion criteria. Full-text articles were assessed for eligibility, resulting in the inclusion of 172 studies in the final review.

Data were extracted using a standardized form, focusing on the following variables: study design, disease type, target organism, biological mechanism, clinical applications, and clinical outcomes. Discrepancies were resolved through discussion among the reviewers.

**Figure 2:**
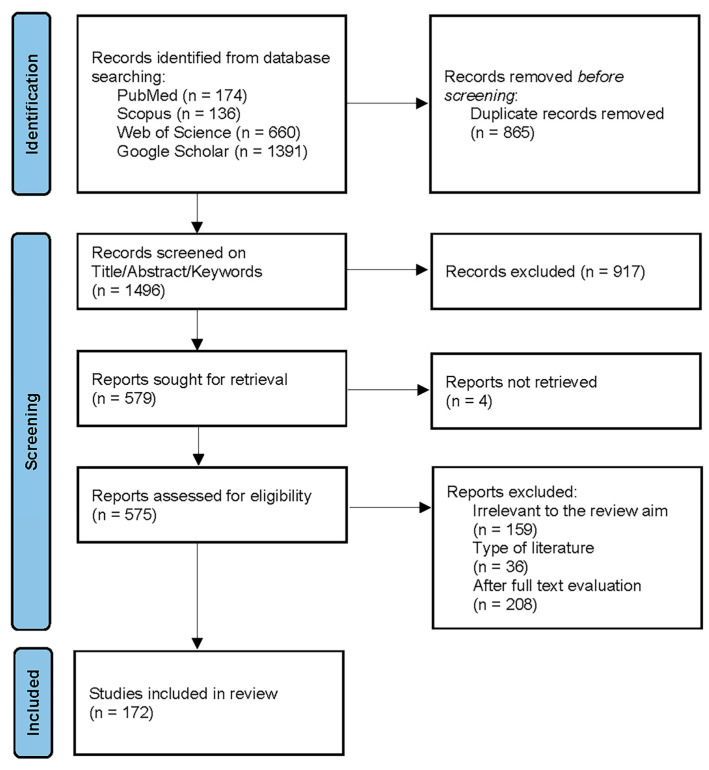
Search flowchart as described in the PRISMA guidelines. Caption: (n = number of records).

The workflow of the paper screening process is reported in Figure 2, according to the “PRISMA 2020 Flow Diagram” [22].

### 2.4. Calibration Process

A calibration exercise was conducted with a random sample of 20 studies to ensure consistency among the three reviewers. Agreement was reached on 90% of the studies before proceeding with the full screening.

## 3. Results

### 3.1. Dental Caries

Dental caries is a chronic infectious disease that affects the hard tissues of the teeth, whose etiology entails intricate microbial interactions. Among cariogenic microorganisms, *S. mutans*, *Lactobacillus* species, and *Actinomyces* species stand out for their robust acidogenic and aciduric capabilities, believed to be closely associated with the initiation and progression of dental caries [23]. The preventive and therapeutic effects of EGCG on dental caries are mainly reflected in its inhibition of bacterial growth and virulence factors, as well as its anti-biofilm activity (Table 1).

#### 3.1.1. *Streptococcus mutans*

*S. mutans*, a facultative anaerobic Gram-positive coccus, is considered one of the primary cariogenic bacteria in the oral cavity. They synthesize organic acids to promote the demineralization of dental hard tissues and inhibit the growth of certain normal bacteria [40]. Additionally, they ferment sucrose and utilize glycosyltransferase to synthesize insoluble extracellular polysaccharides, thereby enhancing adherence to tooth surfaces and promoting the formation of cariogenic biofilms [41]. The anti-caries activity of EGCG against *S. mutans* encompasses multiple aspects, including sugar uptake, acidogenicity, aciduricity, and biofilm formation.

The phosphoenolpyruvate-dependent phosphotransferase system (PEP-PTS) is a group of enzymes involved in transporting sugars into bacterial cells, consisting of enzymes located on the cell membrane and in the cytoplasm. EGCG inhibits the function of PEP-PTS by inhibiting the activity of enolase and the expression of the *eno* gene [32] and by non-competitively binding to the membrane-embedded enzyme II complex, a sugar uptake-related enzyme [26]. It results in a decrease in sugar internalization and suppressed glycolysis, which indirectly leads to reduced acid production by *S. mutans* cells [26,31].

EGCG also directly inhibits bacterial acid production. Han et al. [31] demonstrated that EGCG markedly reduced the production of various organic acids in bacterial culture media, including lactic acid, formic acid, and acetic acid, with the greatest reduction observed in lactic acid levels. EGCG simultaneously inhibits the LDH activity of *S. mutans* at both transcriptional and enzymatic levels [30,32]. This may account for the substantial decrease in lactic acid production.

The acid adaptation of *S. mutans* primarily relies on the F_1_F_0_-ATPase system and the agmatine deiminase system (AgDS). The membrane-bound F_1_F_0_-ATPase system pumps protons out of the cell via proton pumps, while AgDS converts agmatine to putrescine, ammonia, and CO_2_, raising intracellular pH [42]. EGCG inhibits the gene expression and enzymatic activity of F_1_F_0_-ATPase and AgDS, leading to intracellular acidification, which inhibits bacterial metabolism and growth [32].

EGCG showed significant direct activity against biofilm matrices, including inhibiting the formation of amyloid fibers in the matrix [24], inhibiting the formation of acquired enamel pellicle [43], and increasing the proportion of unstructured biofilm [30]. However, the primary mechanism of anti-biofilm activity remains the inhibition of extracellular polysaccharide (EPS), which serves as the framework of biofilms [44]. Glucosyltransferase (GTF) is an enzyme that utilizes sucrose as a substrate to synthesize glucans, the main component of EPS [45]. Xu et al. [33] reported that EGCG at sub-minimum inhibitory concentration (sub-MIC) significantly downregulated the expression of the *gtfB*, *gtfC*, and *gtfD* genes. Schneider-Rayman et al. [35] further found that EGCG significantly downregulated the expression of the *brpA* gene, which regulates biofilm formation. Moreover, the minimum biofilm inhibitory concentration (MBIC) of EGCG against *S. mutans* was significantly lower than the MIC [24,25,28,33,34], further corroborating the anti-biofilm activity of EGCG.

#### 3.1.2. *Lactobacillus* Species and *Actinomyces* Species

The types and quantities of *Lactobacillus* species in the oral cavity are associated with dietary habits, age, and caries status [46]. *Lactobacillus* species cannot colonize on tooth surfaces independently [47]. Therefore, they are generally considered to be mainly involved in the progression rather than the initial occurrence of dental caries.

EGCG exhibits significant inhibitory effects on the growth of various *Lactobacillus* species [29,36] and suppresses their acid production [28], thereby arresting the progression of dental caries. Clinical trials further demonstrated that EGCG or green tea extracts can rapidly reduce the number of *Lactobacillus* with continuous effects [48,49]. However, some studies suggest that *Lactobacillus* may have a potential inhibitory effect on the progression of dental caries [50] and that an appropriate concentration of EGCG in combination with Lactobacillus may exert a synergistic anti-caries effect [29]. It could be due to the differing sensitivities of certain *Lactobacillus* strains and other pathogens to EGCG.

Metagenomic sequencing showed that *Actinomyces* phylum is the most prevalent within deep dentin carious lesions, suggesting a strong association between *Actinomyces* and deep dentin caries [51]. EGCG primarily exhibits its anti-caries properties by inhibiting the growth and adhesion of *Actinomyces* and suppressing the formation of multispecies biofilms on deep dentin surfaces by concurrently inhibiting the growth of various bacteria, including *Actinomyces* and *Enterococcus faecalis* [37,38,52]. However, Wang et al. found that EGCG at concentrations significantly below the MIC paradoxically increased the biofilm formation of *Actinomyces naeslundii* on hydroxyapatite surfaces [39]. This could be due to the induced efflux of Ca^2+^ from *A. naeslundii* cells, which increased their auto-aggregation and consequently their biofilm formation.

EGCG exhibits strong inhibitory activity against major cariogenic bacteria such as *S. mutans*, *Lactobacillus* species, and *Actinomyces* species. It hinders the progression of dental caries by inhibiting bacterial growth, virulence factors, and biofilm formation. Additionally, delivery systems such as nanovesicle in-situ gel [53], chitosan nanoparticles [54], and lipid–chitosan hybrid nanoparticles [55] were developed as novel administration methods for EGCG. Compared to mouthwash, these systems significantly enhance the utilization efficiency and prolong the time of effective concentration of EGCG. They were proven to possess excellent antibacterial and anti-cariogenic properties.

Currently, numerous studies in vivo demonstrated the preventive and therapeutic effects of green tea extracts on dental caries. However, studies specifically investigating the anti-cariogenic activity in vivo of EGCG are quite limited (Table 2). There is a lack of sufficient evidence in vivo supporting the short-term and long-term effects of EGCG on dental caries in the oral cavity. Further research in vivo is needed to address these gaps.

#### 3.1.3. EGCG in Adhesion and Dentin Sealing

Composite resin restoration stands as the primary approach for treating tooth decay. Residual bacteria remaining near the adhesive–hard tissue interface significantly impact bonding strength [58]. The application of antimicrobial agents to delay the decline in bonding strength is crucial to prolonging the lifespan of restorations.

Chlorhexidine is a commonly used chemical antimicrobial agent, but pretreatment with chlorhexidine demonstrates a negative effect on the bonding strength of adhesives to dentin [59]. To clarify the sentence, it could be revised to “In contrast, pretreatment with EGCG doesn’t decrease the dentin bonding strength and it increases the immediate microtensile bond strength (μTBS). Additionally, the μTBS level remains stable after thermal cycling [60,61,62]. The EGCG-incorporating ethanol wet bonding method for bonding adequately wets the dentin surface, significantly reduces nanoleakage, inhibits the formation of *S. mutans* biofilms, and simultaneously deactivates endogenous proteases in the hybrid layer, and preventing damage to dentin collagen [60,63,64]. Zhang et al. [65] improved this method by replacing ethanol with DMSO, reducing solvent evaporation, increasing EGCG concentration, and significantly enhancing aging bond strength, thereby positively impacting long-term bonding stability. In addition to inhibiting the adhesion and growth of cariogenic bacteria, EGCG prevents degeneration and degradation of dentin collagen and degradation of adhesives by inhibiting the activity of matrix metalloproteinases (MMPs) [66,67] and neutralizing potential harmful free radicals [68]. Additionally, with galloyl moiety providing hydrophobicity, hydrogen bonding, and van der Waals forces (Figure 1), EGCG participates in cross-linking between collagen fibers and adhesives, enhancing bonding strength [63].

In the restoration process for dentin caries, to prevent bacterial invasion into pulp tissue through dentinal tubules, it is usually necessary to seal the dentinal tubules in the carious lesions after thorough disinfection. Epigallocatechin-3-gallate-encapsulated nanohydroxyapatite/mesoporous silica nanoparticles (EGCG@nHAp@MSN) almost completely seal dentinal tubules, simultaneously and sustainably release Ca^2+^ and ${\mathrm{PO}}_{4}^{3-}$ to promote remineralization, prevent bacterial invasion, and deactivate MMPs originating from dentin to reduce degradation of dentin collagen up to 30 days [69,70]. EGCG and poly(allylamine)-stabilized amorphous calcium phosphate, a new material with high biocompatibility, not only exhibits antibacterial and remineralization-promoting activities similar to EGCG@nHAp@MSN [71], but also promotes odontoblastic differentiation of human dental pulp stem cells (hDPSCs) and inhibits pulp inflammation [72].

Due to its excellent antibacterial properties, EGCG significantly improves the bonding strength between composite resin and dental tissues in restorative dentistry. Therefore, developing novel drug delivery platforms with sustained-release EGCG and good biocompatibility, as adhesive or pulp capping materials, holds promising application prospects.

### 3.2. Pulpal and Periapical Diseases

Pulpitis and periapical lesions are mainly caused by trauma or infection. Pathogens or their products can invade pulp tissue through dentinal tubules or perforations, triggering inflammatory responses in the pulp [73]. As inflammation worsens, endodontic pathogens spread to the periapical region, inducing inflammation and destruction of periapical tissues [74].

#### 3.2.1. Endodontic Infection

EGCG demonstrated excellent inhibitory effects on various pathogens that cause pulp inflammation. EGCG significantly inhibited the growth of endodontic pathogens, including *S. mutans*, *Fusobacterium nucleatum*, and *E. faecalis*, with an effect comparable to that of glutaraldehyde, but with significantly lower cytotoxicity [75]. Moreover, EGCG inhibits the biofilm formation of these pathogens within root canals and exhibits synergistic effects when used in combination with drugs such as fosfomycin and cationic peptides [37,38].

#### 3.2.2. Effects of EGCG on Pulp and Periapical Tissues

EGCG was shown to directly alleviate pulp inflammation and mitigate the damage caused by pathogens to the pulp and periapical tissues. EGCG reduces the infiltration of inflammatory cells and alleviates tissue inflammation by inhibiting the expression of various inflammatory cytokines at the lesion site, such as interleukin-1 (IL-1), IL-6, IL-8, IL-10, IL-12, IL-17, interferon-γ, and tumor necrosis factor-α (TNF-α) [76,77,78]. Additionally, EGCG reduces the stimulation of human dental pulp cells (hDPCs) by lipopolysaccharide (LPS) and peptidoglycan (PG), lowering the expression and secretion of IL-1β-mediated vascular endothelial growth factor (VEGF) and cyclooxygenasease-2 [78], and inhibiting ROS-induced apoptosis and tissue damage in hDPCs [77,79]. In vitro experiments on mice further confirmed the protective effect of EGCG on pulp tissue, showing that EGCG paste is comparable to calcium hydroxide in inhibiting MMP activity and promoting the recovery of periapical lesions [80].

EGCG facilitates tissue repair and regeneration in the treatment of pulpitis and periapical disease. Stem cells from the apical papilla (SCAPs) are desirable sources of dentin regeneration. EGCG plays a significant role in promoting the osteo-/odontogenic differentiation of SCAPs, as evidenced by increased alkaline phosphatase activity and mineral deposition, along with upregulated expression of osteo-/odontogenic markers [81,82]. Animal experiments demonstrated that EGCG, when used as a dressing in root canals or extraction sockets with periapical lesions, not only alleviates local inflammation, but also significantly promotes fiber formation and bone regeneration in periapical tissues [83,84]. Building on these findings, Cao et al. [85] developed a novel nanoassembly of ECE based on eucommia carbon dots and EGCG, which exhibited promising effects in promoting angiogenesis and enabling dentin differentiation both in vivo and in vitro.

EGCG exhibits strong inhibitory effects on the activity and virulence factors of various pathogens related to pulpal and periapical infections. Additionally, it has low irritability to periodontal and periapical tissues and promotes tissue regeneration, suggesting that it is necessary to design novel root canal sealer materials capable of long-term EGCG release.

### 3.3. Periodontal Diseases

Periodontal disease is a chronic inflammation that occurs in the supporting tissue of the teeth. The microorganisms within dental plaque are initiating factors for periodontal disease, but the composition of microorganisms and host immune responses vary, leading to different severity of periodontal disease [86]. The effects of EGCG on periodontal diseases include the growth and virulence of various periodontal pathogens, alleviating inflammation, and promoting periodontal tissue regeneration.

#### 3.3.1. *Porphyromonas gingivalis*

*P. gingivalis*, a Gram-negative obligate anaerobe, is considered to be the primary pathogen in periodontal diseases, particularly chronic periodontitis. ”It mainly aggregates in dental plaque and express various virulence-associated genes, including genes encoding lipopolysaccharide, collagenase, gingipains, and fimbriae, which directly or indirectly cause damage to periodontal tissues [87].

EGCG exerts a direct inhibitory effect on the growth and adhesion of *P. gingivalis*. At low concentrations, it inhibits bacterial growth and biofilm formation [29,88] and downregulates the gene expression of *hagA* and *hagB,* which regulate bacterial colonization and the gene expression of *fimA*, which encodes type I fimbriae [89]. At higher concentrations, EGCG binds to the bacterial cell membrane, induces the production of ROS, directly damages the cell membrane and cell wall of *P. gingivalis*, and disrupts cellular integrity, thereby exerting bactericidal effects [90].

Furthermore, EGCG inhibits various virulence factors of *P. gingivalis* and its destructive effects on periodontal tissues. Cysteine proteases on the surface of *P. gingivalis* cells, including lysine-specific gingipains (Kgp) and arginine-specific gingipains Rgp), are essential for survival and pathogenicity [91]. Gingipains promote the detachment of *A. actinomycetemcomitans* from biofilms [92], and Kgp regulates biofilm formation by microcolony detachment [93]. EGCG downregulates the gene expression of Kgp and Rgp [89], disrupts the bacterial cytoplasmic membrane, and forms outer membrane vesicles (OMVs), reducing gingipains levels on the membrane and influencing biofilm detachment from the tissue surface [90]. LPS induces macrophage differentiation into osteoclasts in periodontal tissues and induces intracellular oxidative stress, representing another important virulence factor of *P. gingivalis* [94]. EGCG can inhibit the LPS-induced secretion of IL-6 and TNF-α by macrophages and osteoclasts [95]. By suppressing IL-6 secretion, EGCG also inhibits the expression of MMP-1, thereby reducing tissue damage [96]. Additionally, EGCG inhibits the collagenase activity of *P. gingivalis*, thereby suppressing its ability to degrade type I collagen [97]. EGCG significantly and dose-dependently downregulates the expression of the *hem*, the gene encoding hemolysin of *P. gingivalis* [89].

*P. gingivalis* interferes with the normal immune function of the host, inhibiting the defense function of periodontal tissues. Lagha et al. [97] reported that EGCG enhanced the integrity of a gingival keratinocyte monolayer, as shown by the increase in TER and the reduction in FITC-conjugated 4-kDa dextran transport, and protected gingival keratinocyte from the *P. gingivalis*-induced loss of barrier integrity. Additionally, EGCG inhibits the degradation of human β-defensin by *P. gingivalis*, an antimicrobial peptide synthesized by neutrophils, thereby restoring the defensive function of periodontal tissues [98].

To sum up, EGCG inhibits the growth and adhesion of *P. gingivalis*, disrupts biofilm formation, and inhibits gingipains, collagenase, and other virulence factors to alleviate damage to periodontal tissues. Currently, some studies demonstrated the regulatory effects of curcumin on the interactions between periodontal pathogens [93]. Therefore, future research should investigate the impact of EGCG on the interactions between *P. gingivalis* and other periodontal pathogens.

#### 3.3.2. Other Periodontal Pathogens

In addition to *P. gingivalis*, *Aggregatibacter actinomycetemcomitans*, *F. nucleatum*, and *Prevotella intermedia* are also involved in the progression of periodontal disease.

*A. actinomycetemcomitans* is a facultative anaerobic Gram-negative bacterium, mainly detected in aggressive periodontitis patients [99]. Its virulence factors mainly include adherence proteins, polysaccharides, LPS, and toxins, such as cytolethal distending toxin and leukotoxin (LtxA) [100]. EGCG can directly inhibit the growth and viability of *A. actinomycetemcomitans*, but its susceptibility to EGCG is notably lower in comparison to other periodontal pathogens [29]. LtxA can subvert the host immune response by binding to the β2 integrin lymphocyte function-associated antigen-1 on white blood cells, causing cell death [101]. It is the primary virulence factor of *A. actinomycetemcomitans* involved in the rapid progression of periodontitis. EGCG at sub-MIC promotes LtxA production but alters its structure to reduce its affinity for cholesterol on host cell membranes and increase its affinity for bacterial cell membranes, and it reduces the release of OMVs containing LtxA, resulting in reduced cytotoxicity in the culture supernatant [102,103]. Additionally, a single low dose of EGCG did not protect host cells from *A. actinomycetemcomitans*-mediated cytotoxicity. Still, multiple administrations led to a significant increase in the viability of human myeloid leukemia mononuclear cells [102], suggesting that the dosing strategy of EGCG may influence its therapeutic effects. Morin et al. [104] discovered that co-cultures of macrophages and gingival fibroblasts secreted various MMPs with the stimulation of *A. actinomycetemcomitans* LPS, with MMP-3 and MMP-9 being the most abundant. The addition of EGCG led to a dose-dependent reduction in MMP secretion, with MMP-9 showing the most significant decrease. LPS of *A. actinomycetemcomitans* also significantly promotes the secretion of inflammatory cytokines in 3D co-cultures of human gingival fibroblasts (hGFs) and human gingival epithelial cells. EGCG restores cell viability and inhibits the secretion of LPS-induced cytokines such as IL-6, IL-8, interferon-γ inducible protein 10 (IP-10), and granulocyte colony-stimulating factor (G-CSF), thereby alleviating tissue inflammation [105].

*F. nucleatum*, a Gram-negative anaerobic bacterium, serves a structurally supportive role in dental plaque biofilms by bridging primary colonizers to secondary colonizers [106]. Duque et al. [38] found that *F. nucleatum* biofilms were eliminated by EGCG at one-tenth of MIC. Another study demonstrated that EGCG significantly inhibited the adhesion of *F. nucleatum* to oral epithelial cells and collagen fibers, as well as the bacterium’s hemolytic activity and H_2_S production [107].

*P. intermedia* is a Gram-negative obligate anaerobe primarily found in the oral cavity and serves as the primary colonizer in dental plaque biofilms [108]. An animal experiment showed that EGCG directly reduced the activity of *P. intermedia* isolated from periodontal disease dogs [109]. Takuya et al. [29] found that *L. salivarius* WB21 synergistically enhanced the inhibitory effect of EGCG on *P. intermedia*. Additionally, EGCG can alleviate LPS-induced tissue impairments [110].

In summary, EGCG demonstrates significant inhibitory effects on various periodontal pathogens. It suppresses bacterial growth, reduces biofilm formation, and inhibits the damage caused by bacterial virulence factors to periodontal tissues (Table 3). However, there is currently insufficient research to fully elucidate the effects and mechanisms of EGCG on periodontal pathogens and their virulence factors other than *P. gingivalis*, which should be the focus of future studies.

#### 3.3.3. Effects of EGCG on Periodontal Tissues

The antigenic components and virulence factors produced by dental plaque microbiota can directly lead to the destruction of periodontal tissues, as well as induce host immune and inflammatory responses, thereby exacerbating periodontal tissue destruction [114]. In addition to its indirect protective effect on periodontal tissues through bacteriostatic action, EGCG also directly exhibits anti-inflammatory and antioxidant effects, mitigating the damage to periodontal tissues and promoting the regeneration of periodontal tissues [115] (Table 4).

The alleviation of destruction of periodontal tissues by EGCG mainly relies on its inhibition of inflammatory factors and cells. EGCG can maintain cell viability and inhibit the production of various inflammatory cytokines dose-dependently, such as IL-1 and IL-6 [95,96,105,109,110,118,119]. Inflammation cytokines, such as IL-6, increase the expression and activity of several MMPs in periodontal tissues. However, EGCG directly inhibits the recognition and activation of MMPs by chelating Zn^2+^ via B- and D-rings (Figure 1) [65] and suppresses their gene expression and secretion in host cells by blocking the MAPK signaling pathway [104,110]. Tian et al. [120] developed an EGCG-based nanoparticle, which had a significant property of ROS scavenging and protective effects against oxidative stress. Zou et al. [121] found that EGCG reduced orthodontic tooth movement and orthodontic-induced root resorption in rats and was able to attenuate osteoclastogenesis on the pressure side and promote osteogenesis on the tension side.

EGCG also has a strong ability to promote the proliferation of periodontal tissues and differentiation into osteoblasts, facilitating alveolar bone regeneration. EGCG can increase alkaline phosphatase activity in human periodontal ligament cells (hPDLCs) and human alveolar osteoblasts, facilitate the differentiation of hPDLCs into osteoblasts, upregulate the gene expression of the osteogenic biomarkers including OSX, OCN, RUNX2, and BMP2, and promote extracellular matrix mineralization in periodontal tissues [116,117]. Su et al. [88] prepared novel polylactide composite microspheres encapsulated with EGCG and nano-hydroxyapatite, which not only exhibited strong antimicrobial activity against typical periodontal pathogens, but also directly promoted osteogenic differentiation of periodontal ligament stem cells. Additionally, an increased number of M2 macrophages, upregulated expression of growth factors, and promotion of new bone formation in vivo were observed in rats with the application of EGCG-modified collagen membranes for guided bone regeneration [122].

Several clinical studies support the ameliorative effects of EGCG on periodontal inflammation (Table 5). A split-mouth, randomized clinical trial showed that there was a reduction in probing depth and clinical attachment loss, and a significant decrease in bleeding index with the scaling and root planning (SRP) plus EGCG medication compared with SRP alone [123]. Another split-mouth clinical trial supported these results and demonstrated that EGCG prolonged the maintenance of SRP effects and decreased the abundance of *Tannerella forsythia* [124]. Furthermore, multiple clinical trials demonstrated the efficacy of green tea extract in preventing and treating periodontal disease through different delivery systems and administration methods. These methods, primarily involving green tea catechins as the active ingredient, include gels [125,126] and strips [113] placed in periodontal pockets, mouth rinses [127], and brewed green tea consumption [128].

In summary, EGCG alleviates the destruction of periodontal tissues by inhibiting the formation of osteoclasts, synthesizing inflammatory factors, and reducing oxidative stress. Additionally, EGCG demonstrates a robust ability to promote the proliferation of hPDLCs and their differentiation into osteoblasts, thereby facilitating periodontal tissue regeneration. Several clinical studies also confirmed the beneficial effects of EGCG on periodontitis. However, most clinical studies to date focus on the adjunctive role of green tea extracts in the treatment of periodontitis. Further clinical research is needed to ensure its efficacy and safety in periodontal disease treatment.

### 3.4. Nonneoplastic Diseases of the Oral Mucosa

Oral mucosal diseases comprise a diverse spectrum of conditions stemming from malignant, benign, and nonneoplastic processes, with nonneoplastic diseases representing a significant portion of the disease burden [129]. Infection, immune dysregulation, systemic diseases, chemical irritants, and radiation are primary etiological factors contributing to oral mucosal lesions [130].

#### 3.4.1. Oral Leukoplakia and Lichen Planus

Oral leukoplakia is a prevalent and potentially malignant condition of the oral mucosa, with its rate of malignant transformation still debated, ranging from 0.1% to 36.4% [131]. Tao et al. [132] reported that EGCG rapidly induced mitochondria-localized reactive oxygen species in premalignant leukoplakia cells and downregulated the expression of sirtuin 3 (SIRT3), a key regulator of mitochondrial oxidative stress, but not in hGFs. Bioinformatics analysis revealed that EGCG served as an upstream regulatory factor, potentially inhibiting cell malignant transformation by modulating pathways such as epithelial-mesenchymal transition, inflammatory response, and focal adhesion [133].

Lichen planus is an immune-mediated inflammatory condition leading to characteristic lesions on the skin and mucous membranes, and up to 77% of patients with lichen planus have oral disease [134]. EGCG reduces C-X-C motif chemokine ligands (CXCL10) and myxovirus resistance protein 1 staining intensity in epidermis equivalents and CXCL10 secretion by keratinocytes upon stimulation, demonstrating potential for local treatment of lichen planus [135].

#### 3.4.2. Oral Submucous Fibrosis

Oral submucous fibrosis (OSF) is a potentially malignant disorder of the oral cavity, with a high rate of malignant transformation. It is generally believed to be associated with areca nut chewing, consumption of chili, genetic factors, and immune processes [136]. Oral mucosal fibroblasts exposed to irritations such as areca nut alkaloids produce various cytokines, among which transforming growth factor-β (TGF-β) is prominent [137].

EGCG can reduce mucosal fibrosis by inhibiting sphingosine-1-phosphate-induced JNK phosphorylation [138] and TGF-β_1_-induced early growth response-1 in buccal mucosal fibroblasts (BMFs) [139,140], thereby decreasing the synthesis of connective tissue growth factor and the gene expression of type I collagen and collagen protein. Additionally, EGCG inhibits thrombin-activated protease-activated receptor-1 [141] and integrins [142], and arecoline-induced intracellular ROS [143], further suppressing TGF-β_1_ production and BMFs apoptosis. Consequently, Mehta et al. designed an EGCG-loaded mucoadhesive hydrogel with sustained-release capabilities and excellent oral mucosal adhesion properties [144]. This hydrogel significantly improved the degree of mouth opening in a rat OSF model induced by areca nut extract [145] and downregulated the expression of TGF-β1, collagen type-1A2, and type-3A1 mRNA in BMFs in vitro, thereby reducing the risk of malignant transformation [146].

Oral potentially malignant disorders (OPMD) encompass a heterogeneous group of lesions with varying risks of progressing to invasive cancer [147]. In vitro and animal studies demonstrated that EGCG inhibits the malignant transformation of OPMD by modulating epithelial–mesenchymal transition and inflammatory responses. Bioinformatics research identified potential regulatory targets in OPMD, such as oral leukoplakia, and further cellular experiments should be designed to determine the effects of EGCG on these molecular targets.

#### 3.4.3. Oral Mucositis

Chemical irritation and radiation are significant causes of oral mucositis, particularly in cancer patients undergoing chemotherapy or radiotherapy. An in vitro study found that EGCG reduced the cytotoxicity of irinotecan toward oral keratinocytes and epithelial cells by reducing intracellular ROS generation, inhibiting the secretion of IL-6 and IL-8, and restoring irinotecan-induced decrease in the secretion of MMPs [148]. Pan et al. [149] reported that following treatment with EGCG, levels of inflammatory cytokines IL-6 and TNF-α in the mucosa of mice treated with acetic acid significantly decreased, damaged tissues were repaired, and the diversity of oral microbial species increased. A clinical trial found that EGCG demonstrated a significant alleviating effect on radiation-induced mucositis and promoted the repair of oral mucosal injury in head and neck cancer patients [150].

Based on these studies, Shao et al. [151] developed a novel in situ mucoadhesive hydrogel containing EGCG. Animal experiments showed that the novel hydrogel significantly upregulated the expression of cytokeratin 10 and proliferating cell nuclear antigen, reduced the production of inflammatory factors, and promoted mucosal repair.

In general, EGCG alleviates medication- and radiation-induced oral mucositis primarily by inhibiting the synthesis and secretion of inflammatory cytokines. Novel hydrogel materials incorporating EGCG also demonstrated significant anti-inflammatory effects and the ability to promote mucosal repair. Therefore, developing more biocompatible materials and evaluating their role as adjunctive treatments for head and neck cancer patients undergoing radiotherapy and chemotherapy should be key research directions in the future.

### 3.5. Salivary Gland Diseases

Salivary gland diseases encompass a spectrum of conditions with various etiologies, including sialadenitis, developmental anomalies, cysts, and tumors. Viruses, bacteria, and autoimmune responses are the primary causes of sialadenitis, including mumps, sialolithiasis, and Sjögren’s syndrome [152]. Tumors occurring in the salivary glands typically originate from glandular epithelium, with benign tumors being predominant [153].

#### 3.5.1. Sjögren’s Syndrome

Sjögren’s syndrome (SS) is characterized as a systemic autoimmune rheumatic disease affecting exocrine glands, such as lacrimal and salivary glands, leading to dry eye and dry mouth, particularly prevalent in women over the age of 40 [154,155]. Saito et al. [156] EGCG altered the gene expression levels of 11 sialadenitis-related molecules, including heme oxygenase-1, improving salivary gland damage in mice with autoimmune sialadenitis. Additionally, EGCG reduced ROS levels in the tissues, thereby relieving ROS-mediated inhibition of water channel aquaporin 5 gene expression and increasing salivary flow [157]. A clinical trial exhibited that a natural formulation containing tea catechins significantly increased unstimulated (3.8-fold) and stimulated (2.1-fold) saliva output compared to baseline, partially restoring salivary function [158].

#### 3.5.2. Medication- and Radiation-Induced Salivary Gland Dysfunction

Patients with head and neck malignancies often require chemotherapy or radiotherapy, which can induce damage to normal salivary gland cells, leading to salivary gland dysfunction [159]. Choi et al. [160] found that EGCG significantly improved saliva flow rate and saliva retention time in mice with radioactive iodine-induced sialadenitis, with effects comparable to the standard antioxidant amifostine. Histological analysis revealed that, following EGCG administration, the mice exhibited more mucin-rich parenchyma and less periductal fibrosis in the submandibular gland, along with significantly reduced cell apoptosis in acini and ducts. Sulistiyani et al. [161] found that EGCG promoted the proliferation of salivary gland epithelial cells and the development of pro-acinar buds and ducts while increasing the populations of epithelial progenitors in buds and ducts and pro-acinar cells. Taha et al. [162] reported that green tea extract blocked methotrexate and induced cytotoxicity in the submandibular salivary glands of rats.

These results suggest that EGCG may inhibit apoptosis of salivary gland epithelial cells and promote their proliferation through its antioxidant activity, thereby alleviating damage to the salivary glands caused by radiation or medication.

#### 3.5.3. Salivary Gland Tumors

Salivary gland tumors are common tumors in the oral and maxillofacial region, with a history of radiation, diet, and other malignancies being important risk factors [153]. Park et al. [163] found that EGCG inhibited the expression of β1 integrin, reducing the expression and enzyme activity of MMP-2 and MMP-9,and providing molecular evidence for the inhibitory effect of EGCG on salivary gland cancer metastasis. Weng et al. reported [164] that EGCG could inhibit proliferation and promote apoptosis of adenoid cystic carcinoma cells by reducing the expression of EGFR, downregulating Bcl-2, and upregulating Bax.

In a word, EGCG alleviates inflammatory damage to the salivary glands by inhibiting inflammatory cytokines and reducing oxidative stress, and it promotes the proliferation and repair of salivary gland epithelium. Additionally, EGCG inhibits the metastasis of salivary gland cancer by downregulating the expression of MMPs and integrins. However, there are currently limited studies on the effects of EGCG on salivary gland diseases, and more research is needed to explore its impact on these conditions.

### 3.6. Oral Cancer

Oral cancer refers to the squamous cell carcinoma occurring in the oral mucosa, accounting for approximately 90% of malignant tumors in the oral cavity. The risk factors of oral cancer mainly include tobacco, alcohol, areca nut, excessive sunlight exposure, and human papillomavirus (HPV) [165,166]. A variety of evidence demonstrated the anticancer activity of EGCG against oral cancer and exhibited synergistic effects with various drugs to enhance the therapeutic efficacy of conventional treatments (Table 6).

#### 3.6.1. Proliferation and Apoptosis

EGCG can regulate signaling pathways and induce oxidative stress in various cancer cells, inhibiting cancer cell proliferation and inducing apoptosis [179]. EGCG induces extracellular ROS and selectively induces intracellular ROS in OSCC cells, significantly inhibiting their proliferation and inducing apoptosis. Additionally, EGCG downregulates the expression of genes associated with oxidative stress, such as metallothionein 3 and SOD, in OSCC cells, while upregulating these genes in hGFs [132,170]. EGCG suppresses OSCC cell proliferation and promotes apoptosis by regulating TAZ or mTOR pathways to inhibit Akt phosphorylation [168,169,180], promoting the JNK/MAPK pathway to enhance phosphorylation of JNK, ERK, and p38 [173], and blocking the Notch signaling pathway [181]. Furthermore, EGCG increases the sensitivity of OSCC cells to chemotherapeutic agents such as 5-FU [175] and vincristine sulfate (VCR) [182], promoting apoptosis and reducing VCR toxicity, thereby extending survival time in mice. EGCG also exhibits synergistic effects with resveratrol [180] and curcumin [183], resulting in decreased expression of p53 and Ki67, reduced levels of survival proteins, and improved clinical efficacy in inhibiting proliferation and promoting apoptosis of OSCC cells.

#### 3.6.2. Invasion and Metastasis

The degradation of extracellular collagen and the formation of invadopodia are crucial mechanisms underlying tumor invasion and metastasis [184]. EGCG inhibits the expression of MMPs in OSCC cells by suppressing Src phosphorylation, inhibiting the activities of RhoA and MEK, and directly reducing MMP activity [167,174,177]. It results in the inhibition of extracellular matrix degradation. Hwang et al. [167] reported that EGCG suppressed functional invadopodia formation, thereby inhibiting cancer invasion in 3D culture and reducing tumor volume and regional infiltration near the stroma in mice.

#### 3.6.3. HPV

HPV is one of the major etiological factors of oral cancer. The incidence of HPV-associated oropharyngeal squamous cell carcinoma is rapidly increasing and surpassed cervical cancer to become the most common HPV-induced cancer in developed countries [185]. EGCG exhibits strong anti-HPV-2 activity and restores the mRNA and protein expressions of the type I interferon signaling pathway suppressed by E7 [186]. Yap et al. [187] reported that EGCG downregulated the expression levels of HPV-18 E6 and E7 proteins in cells, but this was not through affecting E6 and E7 mRNA transcription. Instead, it promoted the degradation of E6 and E7 proteins via the ubiquitin-proteasome pathway.

#### 3.6.4. Clinical Research

Although the inhibitory effect of EGCG on cancer cells was demonstrated in multiple experiments, there is still a lack of convincing in vivo studies, especially clinical research, to prove the benefits of EGCG in cancer treatment (Table 7).

A double-blind, randomized preliminary study suggested that the combination of EGCG and curcumin synergistically improved clinical symptoms, with good patient tolerance. After three months of combination therapy, significant downregulation of various biomarkers of cancer, such as p53, Ki67, and cyclin D1, was observed [183]. A phase I b study showed that chemoprevention with green tea polyphenol and erlotinib achieved a high rate of pathologic response with excellent cancer-free survival. They also observed statistically insignificant reductions in ERK and Ki67 [188].

In a single-blind randomized controlled trial by Liao et al. [189], the administration of a mouthwash containing green tea extract significantly improved oral health in patients with oral cancer undergoing radiotherapy and chemotherapy. Zhu et al. [150] also reported that using EGCG mouthwash during radiotherapy significantly reduced radiation-induced oral mucosal injury and improved patient satisfaction. Patients using green tea mouthwash showed significant improvement in oral mucosal health, enhancing their willingness to adhere to cancer treatment.

A multicenter case-control study conducted in China included 723 cases and 857 controls, finding a significant correlation between green tea intake and reduced risk of oral cancer in males, with a stronger correlation observed in male smokers [190]. In a Japanese prospective cohort study, researchers found that subjects who drank five or more cups of green tea had a lower risk of oral cancer, with a greater reduction observed in females, although this result was not statistically significant [191]. However, clinical studies conducted in Italy [192] and the United States [193] concluded that tea consumption did not benefit to preventing oral cancer.

According to a meta-analysis by the Cochrane Collaboration of 142 studies involving more than 1.1 million participants, different evidence regarding the consumption of green tea and its potential to reduce the risk of cancer is conflicting [16]. This contradiction may be due to the different types of tea consumed by the population in various studies, as green tea contains catechins, while black tea primarily contains theaflavins and thearubigins, which have different biological activities [194]. The low bioavailability of tea polyphenols in tea consumption may also be a significant reason for the contradictory results between experimental and observational studies.

In summary, results from animal and cell studies demonstrate the inhibitory effects of EGCG on the proliferation, metastasis, invasion, and angiogenesis of oral cancer. It appears that EGCG may have preventive and regressive effects on oral malignant tumors. However, there are few clinical studies investigating the preventive and therapeutic effects of EGCG on oral cancer. The existing studies included an insufficient number of patients, and some clinical results are contradictory. Robust evidence supporting the application of EGCG in the field of oral cancer is still lacking. Its benefits to humans remain inconclusive and require further epidemiological and clinical research.

## 4. Discussion and Prospects

EGCG exhibits inhibitory effects on various major pathogenic microorganisms responsible for oral infectious diseases, such as caries, periodontal disease, and pulpal and periapical diseases. EGCG at low concentrations primarily exert antibacterial effects by inhibiting virulence factors and effectively inhibiting the formation and maturation of bacterial biofilms EGCG at higher concentrations can directly induce the production of H_2_O_2_ in cell membranes, disrupting microbial cell structures and exerting bactericidal effects.

Elevated levels of ROS were observed in the diseased tissues of periodontal disease, Sjögren’s syndrome, and mucositis. EGCG can directly scavenge excessive ROS and inhibit their production and accumulation by regulating signaling pathways and enhancing antioxidant enzyme activity, thereby preventing damage to cells. However, due to its high reactivity, EGCG at a higher concentration may undergo auto-oxidation reactions, leading to the generation of ROS, which is also an important mechanism for EGCG-induced apoptosis of tumor cells [195].

Inflammation reactions induced by stimulation such as microorganisms, radiation, or trauma are the main mechanisms of tissue damage in pulpitis, periodontitis, mucositis, and sialadenitis. EGCG regulates molecular signaling pathways to inhibit the secretion of inflammatory factor, alleviating inflammatory tissue damage.

EGCG exhibits anti-tumor activity against various cancers. Results from animal and cell studies also demonstrate the significant inhibitory effects of EGCG on the proliferation, metastasis, invasion, and angiogenesis of oral cancer. However, the results of several clinical studies currently show contradictions, and there is still a lack of strong evidence supporting the application of EGCG in the field of oral cancer.

Bioavailability and biosafety are critical issues in the clinical appliance of EGCG. The accessibility of EGCG to specific body tissues is essential to achieve a satisfactory therapeutic effect. However, the bioactivity of EGCG relies on multiple phenolic hydroxyl groups in its molecule, leading to low stability in alkaline and neutral media and low lipid solubility. These properties result in low membrane permeability, high oxidative degradation, and rapid metabolic transformations [196]. Additionally, EGCG is carried via passive diffusion across the intestinal epithelium due to its hydrophilic nature and lack of specific receptors. The efflux transport system actively transports intracellular EGCG back to the extracellular intestinal space, further limiting its absorption [197]. However, the application of high-concentration EGCG carries a higher risk of toxicity, especially in systemic administration. Animal experiments showed that daily feeding of 500 mg·kg^−1^ EGCG had no adverse effects on rats, whereas feeding 2000 mg·kg^−1^ EGCG per day led to rat deaths within 72 h [198,199,200]. A systematic review conducted in 2018 examined adverse event (AE) data from 159 human intervention studies. It found that concentrated, catechin-rich green tea preparations resulted in hepatic AEs in a dose-dependent manner when ingested in large bolus doses. However, no such adverse effects were observed when green tea was consumed as brewed tea or extracts in beverages or as part of food [201]. A clinical trial reported that a daily dose of 800 mg of EGCG alone was well-tolerated in reproductive-aged women [202]. Additionally, another trial reported a daily dose of 10 mg/kg of EGCG for children with Down syndrome did not increase the risk of severe adverse events or the incidence of adverse events related to safety biomarkers [203]. Based on previous experimental results, the European Food Safety Authority (EFSA) set the maximum daily intake of EGCG at 800 mg. Additionally, the EFSA indicated that even if the daily intake is less than 800 mg, potential harm to the human body should not be ruled out due to limited data on the dose–response relationship between EGCG content and abnormal liver parameters [204]. Due to the low concentration, low absorption, and high conversion speed of catechins during tea consumption, green tea, when consumed as brewed tea or extracts in beverages or foods, does not exhibit AEs. However, excessive consumption of green tea can lead to an intake of too much caffeine, affecting neural activity, reducing iron absorption, promoting bone loss [205,206], and causing staining of teeth [207] and dental restorations [208]. Moreover, prolonged consumption of excessively hot tea significantly increases the risk of esophageal [209] and gastric cancer [210]. In summary, drinking 1–2 cups (around 250–500 mL) of moderately warm, light green tea daily is a better choice for health.

Chemical modification of EGCG, reliable delivery systems, in combination with other drugs appear to be feasible strategies to enhance the therapeutic potential of EGCG. The methacrylate polymer formed by the reaction of EGCG and methacryloyl chloride exhibit high chemical stability and can release EGCG steadily over 30 days. The methacrylic acid groups modified on the phenolic hydroxyl groups of EGCG enhance its antibacterial activity against oral pathogens such as *S. mutans* [211]. EGCG-capped gold nanoparticles demonstrate excellent anti-resorptive properties and can carry EGCG into cells, increasing its bioavailability and achieving sustained release to prevent local EGCG accumulation. Additionally, EGCG can promote the cellular uptake of gold nanoparticles [212]. In the treatment of oral cancer, the combined use of 5-FU and EGCG mitigates the impact of medicine on the viability of normal cells and lowers cancer cell survival and migration rates. These effects may be attributed to EGCG altering the cell cycle distribution, preventing cells from developing proper migration abilities [175]. Additionally, the combination of EGCG and vincristine sulfate in preclinical treatments showed more significant inhibition of tumor growth, angiogenic activity, and VEGF expression in xenograft nude mice inoculated with KBV200 cells compared to vincristine sulfate alone [182]. The synergistic antimicrobial potential of EGCG and cationic peptides was also demonstrated on single- and dual-species biofilms associated with endodontic infection [37]. In general, the bioavailability of EGCG can be improved through chemical modifications such as esterification, methylation, or glycosylation, or by optimizing administration methods and delivery systems, such as topical administration or nano-encapsulations to enhance targeting. These approaches can also reduce the risk of toxicity due to local accumulation of EGCG [213].

## 5. Limitations

The writers identified primary search terms before conducting searches in mainstream databases such as Web of Science and PubMed. Despite using controlled vocabularies, synonyms, and alternate spellings, it is possible that some studies or findings were not comprehensively retrieved or were missed. Additionally, this review focused on recent research concerning the role of EGCG in oral diseases. EGCG, being a chemically unstable component of green tea catechins, gained widespread attention relatively late. Therefore, the review primarily included literature published within the last decade. Selected publications from 2010 to 2014 were included, while publications before 2010 were largely omitted.

## 6. Conclusions

Currently, numerous studies, particularly in vitro research, adequately elucidated the antimicrobial, antioxidant, anti-inflammatory, and antitumor effects of EGCG in many oral diseases, including dental caries, periodontal diseases, and oral cancer. The underlying biological mechanisms were explored, highlighting the significant potential of EGCG as a natural therapeutic agent for treating oral diseases. However, there is still a lack of sufficient clinical evidence for the effectiveness of EGCG in most oral diseases, such as mucositis and sialadenitis. Considering the openness of the oral environment and the complexity of host immunity, some significant changes observed in vitro may not necessarily have similar effects in vivo. Whether EGCG can produce clinically significant biological effects in the human body still requires validation through more in vivo experiments. Additionally, the low bioavailability of EGCG and its unclear toxicity mechanisms further complicate its clinical application. Therefore, exploring the combined application of EGCG with other drugs, developing new EGCG derivatives, and designing novel sustained-release delivery systems to achieve higher bioavailability or therapeutic synergistic effects holds great promise. Extensive clinical research is still needed before EGCG can be widely applied in the clinical treatment of oral diseases.

## Figures and Tables

**Table 1:** The effects of EGCG on caries-related bacteria in in vitro studies.

Bacteria	Strain	Intervention	Relevant Findings	Reference, Year
*Streptococcus mutans*	ATCC 700610 (UA159)	50, 100, and 200 μM of EGCG	Inhibited growth and decreased biofilm formation.	[24], 2019
	BCRC 10793	20, 100, and 500 μM of EGCG	Weakly inhibited growth and decreased biofilm formation.	[25], 2021
	NCTC 10449	1 mg·mL^−1^ of EGCG	Inhibited growth, bound to EIIC, inhibited the PEP-PTS activity, enhanced the inhibitory effects of KF, and inhibited acid production.	[26], 2023
	ATCC 700610	0.25 mg·mL^−1^ of EGCG	Decreased CSC- and nicotine-induced biofilm formation.	[27], 2021
	ATCC 25175	0–1000·μg·mL^−1^ of EGCG	Inhibited growth, inhibited acid production, decreased biofilm formation, and downregulated gene and protein expression of GTF.	[28], 2018
	JCM 5705 and JCM 5175	75–300·μg·mL^−1^ of EGCG	Inhibited growth and synergistic actions with *Lactobacillus salivarius.*	[29], 2019
	ATCC 700610	4 mg·mL^−1^ of EGCG	Inhibited acid production, decreased soluble and insoluble polysaccharides, and increased unstructured biofilm.	[30], 2024
	NCTC 10449	0.5, 1, and 2 μg·mL^−1^ of EGCG	Inhibited growth dose-dependently, inhibited acid production, inhibited the PEP-PTS activity, and promoted aggregation.	[31], 2021
	ATCC 700610	31.25–125 μg·mL^−1^ of EGCG	Inhibited growth, inhibited acid production, inhibited the PEP-PTS activity, inhibited the F_1_F_0_-ATPase activity, inhibited the AgDS activity, and downregulated gene expressions of *atpD*, *eno*, *ldh*, and *aguD.*	[32], 2011
	ATCC 700610	7.8–31.25 μg·mL^−1^ of EGCG	Inhibited initial attachment dose-dependently, insignificantly promoted aggregation, and downregulated gene expression of GTF.	[33], 2012
	MTCC 890	0.125–2 mg·mL^−1^ of EGCG	Inhibited growth, inhibited the GTF activity, and decreased biofilm formation.	[34], 2020
	ATCC 700610	0.55–4.4 mg·mL^−1^ of EGCG	Inhibited growth, downregulated the gene expressions of *nox* and *sodA*, inhibited the gene expression and activity of GTF, and decreased biofilm formation dose-dependently.	[35], 2021
*Streptococcus sobrinus*	BCRC No. 14757	20, 100, and 500 μM of EGCG	Weakly inhibited growth and decreased biofilm formation.	[25], 2021
*Streptococcus sanguinis*	JCM 5708	0.5, 1, and 2 μg·mL^−1^ of EGCG	Inhibited acid production and promoted aggregation.	[31], 2021
*Streptococcus gordonii*	JCM 12995	0.5, 1, and 2 μg·mL^−1^ of EGCG	Inhibited acid production and promoted aggregation.	[31], 2021
*Streptococcus salivarius*	JCM 5707	0.5, 1, and 2 μg·mL^−1^ of EGCG	Inhibited acid production and slightly promoted aggregation.	[31], 2021
*Lactobacillus casei*	isolated from a bottle of Yakult^®^	0–1000 μg·mL^−1^ of EGCG	Inhibited growth and inhibited acid production.	[28], 2018
	ATCC 6538	1 mg·mL^−1^ of EGCG	Inhibited growth.	[36], 2023
*Lactobacillus salivarius*	WB21	25 mg·mL^−1^ of EGCG	Inhibited growth.	[29], 2019
*Actinomyces israelii*	ATCC 12102	0.00781–1 mg·mL^−1^ of EGCG, combination with peptide LL-37	Inhibited growth and decreased biofilm formation.	[37], 2021
	ATCC 12102	0.3125–2.5 mg·mL^−1^ of EGCG, combination with fosfomycin	Inhibited growth and decreased biofilm formation.	[38], 2023
*Actinomyces naeslundii*	ATCC 51655	62.5 μg·mL^−1^ of EGCG	Promoted aggregation and biofilm formation.	[39], 2020

EGCG, epigallocatechin gallate; M, mol·L^−1^; KF, potassium fluoride; EIIC, membrane-embedded enzyme II complex; PEP-PTS, phosphoenolpyruvate-dependent phosphotransferase system; CSC, cigarette smoke condensate; GTF, glucosyltransferase; AgDS, agmatine deiminase system; *atpD*, α subunit of the proton translocator; *eno*, enolase; *ldh*, lactate dehydrogenase; *aguD*, antiporter of AgDS; *nox* and *sodA*, genes involved in the protection against oxidative stress.

**Table 2:** The anti-caries effects of green tea extract or EGCG in in vivo studies.

Study Type	Country	Subject	Sample Size	Intervention	Analysis	Relevant Findings	Reference, Year
Double-blind RCT	Brazil	children aged 5–12 years at high caries risk	47	4000 μg·mL^−1^ of EGCG, rinse 1 min.	Cultured saliva	Significant decrease in *Streptococcus mutans* and *lactobacilli*, weaker than CHX and stronger than green tea extract.	[48], 2020
RCT	Italy	Adolescents aged 12–18 years	66	40 mL infusion with 1.6 g green tea, rinse 3 times a day for 7 days.	Cultured saliva	Significant decrease in *Streptococcus mutans* and *lactobacilli.*	[49], 2011
Single-blind RCT	Saudi Arabia	Children aged 4–5 years	40	8 mL infusion with 0.08 g green tea, rinse twice a day for 4 weeks.	Cultured saliva	Significant decrease in *Streptococcus mutans.*	[56], 2019
Double-blind RCT	Iran	Children aged 4–6 years	90	1 mL gels with 5% green tea powder randomly applied on all the teeth at the same coachman position.	Cultured saliva, qPCR	Significant decrease in *Streptococcus mutans* in a week.	[57], 2023
RCT	Iraq	Adults aged 19–23 years	15	15 mL distilled water with 3.75 mg green tea extract and 117.3 mg *Salvadora persica*, twice a day for 4 days.	Cultured saliva, qPCR, recorded plaque quality	Significant decrease in *Streptococcus mutans*, *Streptococcus sanguinis*, *Actinomyces viscosus*, and *Actinomyces naeslundii.*	[52], 2020

RCT, randomized controlled trial; EGCG, epigallocatechin gallate; CHX, chlorhexidine; qPCR, quantitative polymerase chain reaction.

**Table 3:** The effects of EGCG on periodontal pathogens in in vitro studies.

Bacteria	Strain	Intervention	Relevant Findings	Reference, Year
*Porphyromonas gingivalis*	ATCC 33277 and JCM 8525	100–1000 μg·mL^−1^ of EGCG	Inhibited growth and biofilm formation and a synergistic action with *Lactobacillus salivarius.*	[29], 2019
	381	10–5000 μg·mL^−1^ of EGCG	Inhibited growth, damage to the cell membrane and cell wall, and decreased ATP in cells and biofilm formation dose-dependently.	[90], 2014
	ATCC 33277	Microspheres containing 2–8% EGCG	Inhibited growth with 6% EGCG, while promoted growth with pure microspheres.	[88], 2023
	ATCC 33277, HW24D1, and W83	15.6–2000 μg·mL^−1^ of EGCG	Inhibited growth and adherence to epithelial cells, downregulated *hagA*, *hagB*, *rgpA*, *kgp*, *hem*, and *fimA* gene expression, and increased *htrA* gene expression.	[89], 2016
	W83	5.86–187.5 μg·mL^−1^ of EGCG	Inhibited growth and production of CH_3_SH in sub-MIC, and downregulated *mgl* gene expression.	[111], 2010
	ATCC 33277	25–300 μg·mL^−1^ of EGCG	Inhibited infection-induced degradation of hBD.	[98], 2014
	ATCC 33277	15.625, 31.25 and 62.5 μg·mL^−1^ of EGCG	Reduced infection-mediated loss of keratinocyte barrier integrity and decrease in TER, and inhibited gingipains and collagenase activity and bacterial migration.	[97], 2018
	A7436	5, 10, 20, 40 µM of EGCG	Insignificant maintaining of methylation of the promoters induced by *P. gingivalis.*	[112], 2020
*Aggregatibacter actinomycetemcomitans*	JCM 8577	10 mg·mL^−1^ of EGCG	Inhibited growth.	[29], 2019
	ATCC 29522	1–50 μg·mL^−1^ of EGCG	Alleviated LPS-induced inflammation.	[105], 2015
	JP2	5–50 μg·mL^−1^ of EGCG	Increased production of LtxA, while decreased release of OMVs containing LtxA.	[102], 2021
	JP2 and AA704	5–50 μg·mL^−1^ of EGCG	Increased production of LtxA, and promoted affinity of LtxA to bacterial cell surface.	[103], 2020
	ATCC 29522	7.81–125 μg·mL^−1^ of EGCG	Alleviated LPS-induced inflammation.	[104], 2017
*Fusobacterium nucleatum*	JCM 8532	2.5 mg·mL^−1^ of EGCG	Inhibited growth.	[29], 2019
	ATCC 25586	50–1000 μg·mL^−1^ of EGCG	Inhibited growth, adherence, and biofilm formation, chelated iron, inhibited hemolysis, and decreased production of H_2_S.	[107], 2017
	ATCC 25586	7.9–500 μg·mL^−1^ of EGCG	Alleviated LPS-induced tissue impairs.	[110], 2016
*Prevotella intermedia*	ATCC 25611	2.5 mg·mL^−1^ of EGCG	Inhibited growth.	[29], 2019
	ATCC 25611	1 mg·mL^−1^ of EGCG	Inhibited growth and biofilm formation.	[113], 2002
	BX5, isolated from dogs	1 mg·mL^−1^ of EGCG	Inhibited growth.	[109], 2023

EGCG, epigallocatechin gallate; sub-MIC, sub-minimum inhibitory concentration; *hagA*, the gene involved in host colonization; *hagB*, the gene involved in host colonization; *rgpA*, Arg-gingipain A; *kgp*, Lys-gingipain; *hem*, hemagglutinins; *fimA*, type I fimbriae; *htrA*, the gene involved in resistance to oxidative stress; *mgl*, the gene encoding L-methionine-α-deamino-γ-mercaptomethane-lyase; hBD, human β defensin; TER, transepithelial electrical resistance; M, mol·L^−1^; LPS, lipopolysaccharide; LtxA, leukotoxin; OMVs, outer membrane vesicles.

**Table 4:** The effects of EGCG on periodontal tissues in in vitro studies.

Cell Line	Intervention	Other Treatments	Relevant Findings	Reference, Year
hGF C165	10–100 μM of EGCG	1–50 μg·mL^−1^ LPS of *Porphyromonas gingivalis*	Kept cell viability, and decreased LPS-induced secretion of TNF-α.	[95], 2021
hGF from 3 patients	10–50 μM of EGCG	0.1–10 μg·mL^−1^ LPS of *Porphyromonas gingivalis*	Downregulated LPS-enhanced gene expression of MMP-1 and IL-6.	[96], 2014
hGE OBA-9 and hGF ATCC CRL-2014	1 and 5 μg·mL^−1^ EGCG, combined with 0.1 and 0.2 μM of LL-37	3D co-culture, 1 μg·mL^−1^ LPS of *Aggregatibacter actinomycetemcomitans*	Kept cell viability, decreased LPS-induced secretion of G-CSF, GRO-α, IL-6, IL-8, and IP-10, and synergistically decreased MCP-1.	[105], 2015
hGF ATCC CRL-2014 and human monoblastic leukemia cell ATCC CRL-1593.2	7.81–125 μg·mL^−1^ EGCG	3D co-culture, 10:1 ratio to mimic slight inflammation and 1:10 ratio to mimic severe inflammation, 1 μg·mL^−1^ LPS of *Aggregatibacter actinomycetemcomitans*	Decreased LPS-enhanced secretion of MMP-3, MMP-8, MMP-9, greater in 10:1 ratio model.	[104], 2017
hGE B11	25–300 μg·mL^−1^ EGCG	*Porphyromonas gingivalis* ATCC 33277	Increased gene expression and secretion of hBD1 and hBD2 via p38 MAPK and ERK1/2, and inhibited infection-induced degradation of hBD.	[98], 2014
human gingival keratinocyte cell B11	15.625–62.5 μg·mL^−1^ EGCG	*Porphyromonas gingivalis* ATCC 33277	Increased TER, enhanced the function of keratinocyte barrier, and maintained keratinocyte barrier integrity by increasing ZO-1 and occluding.	[97], 2018
THP-1 cell	5–50 μg·mL^−1^ of EGCG	*Aggregatibacter actinomycetemcomitans* JP2	Keep cell viability from LtxA, greater with the multiple-dosing strategy.	[102], 2021
primary hGE	10 μM of EGCG	*Porphyromonas gingivalis* A7436	Prevented infection-induced impairment of epithelial barrier function, induced gene expression, and inhibited infection-induced decrease in abundance of the cell–cell junction proteins.	[112], 2020
human mono-blastic leukemia cell U937 3xκB-LUC	7.9–500 μg·mL^−1^ of EGCG	*Fusobacterium nucleatum* ATCC 25586	Inhibited NF-κB activation, decreased secretion of IL-1β, IL-6, IL-8, and TNF-α dose-dependently, decreased secretion of MMP-3 and MMP-9, and inhibited the secretion and shedding of sTREM-1.	[110], 2016
hAOB and hPDLC isolated from 10 healthy human	0.1–100 μM of EGCG	PI3K-specific inhibitors	Increased ALP activity and mineralized nodules dose-dependently, upregulated gene expressions of RUNX2, BMP2, OSX, and OCN, and activated the PI3K/Akt signaling pathway.	[116], 2021
hPDLC isolated from human healthy premolars	2–10 μM of EGCG	\	Inhibited cell proliferation with 6–10 μM EGCG, increased intracellular ROS dose-dependently with 6–10 μM EGCG, increased ALP activity with 2–4 μM EGCG, increased degree of mineralization, and upregulated gene expressions of COL_1_, RUNX2, OPN, and OSX.	[117], 2019

hGF, human gingival fibroblast; LPS, lipopolysaccharide; EGCG, epigallocatechin gallate; M, mol·L^−1^; TNF-α, tumor necrosis factor-α; MMP, matrix metalloproteinase; IL, interleukin; hGE, human gingival epithelial cell; 3D, three-dimensional; G-CSF, granulocyte colony-stimulating factor; GRO-α, CXC-chemokine ligand 1; IP-10, interferon-γ inducible protein 10; MCP-1, monocyte chemoattractant protein-1; hBD, human β defensin; TER, transepithelial electrical resistance; MAPK, mitogen-activated protein kinase; ERK, extracellular signal-regulated kinase; ZO-1, zonula occludens-1; THP-1 cell, human myeloid leukemia mononuclear cell; LtxA, leukotoxin; NF-κB, nuclear factor kappa-B; sTREM-1, soluble triggering receptor expressed on myeloid cells-1; hAOB, human alveolar osteoblast; hPDLC, human periodontal ligament cell; PI3K, phosphoinostitide 3-kinase; ALP, alkaline phosphatase; RUNX2, runt-related transcription factor 2; BMP2, bone morphogenetic protein 2; OSX, osterix; OCN, osteocalcin; Akt, protein kinase B; ROS, reactive oxygen species; COL_1_, type I collagen; OPN, osteopontin.

**Table 5:** The effects of green tea extract or EGCG on periodontal diseases in in vivo studies.

Study Type	Country	Subject	Sample Size	Intervention	Analysis	Relevant Findings	Reference, Year
Double-blind, split mouth RCT	China	Patients with CP, PPD ≥ 5 mm.	15	Replaced distilled water in the ultrasonic scaler with 5 mg·mL^−1^ of EGCG.	Probing	Insignificant improvement of PPD, CAL, GI, and PI, significant improvement of BI in 12 weeks.	[123], 2022
Double-blind, split-mouth RCT	China	Patients with CP, CAL loss in > 30% of sites.	20	Replaced distilled water in the ultrasonic scaler with 5 mg·mL^−1^ of EGCG.	Probing and cultured subgingival plaque	Improvement of PPD, and decreased abundance of *Tannerella forsythia* in 6 months.	[124], 2021
Single-blind RCT	Thailand	Patients with CP, PPD of 5–10 mm.	21	Placed gels containing over 9.6% *w*/*w* green tea catechins into the pocket, repeatedly applied at 1 and 2 weeks later.	Probing	Insignificant improvement of PPD, CAL, and GI, significant improvement of BI.	[125], 2016
Single-blind, split-mouth RCT	India	Patients with CP, PPD of 4–6 mm in >30% of sites.	30	Placed gels containing over 10 mg·mL^−1^ green tea extract into the pocket.	Probing	Improvement of GI, PPD, and rCAL in 4 weeks.	[126], 2013
Double-blind RCT	Iran	Patients with CP, ≥2 teeth with PPD ≥ 5 mm in each quadrant, CAL > 3 mm.	30	Drank green tea after brushing, twice a day for 6 weeks.	Probing	Insignificant improvement of PI, significant improvement of PPD and BI.	[128], 2018
Double-blind RCT	India	Patients with at least 20 teeth aged 18–60 years, PI ≥ 1.5, GI ≥ 1.0.	110	10 mL mouthwash containing 2% green tea extract for 1 min rinsing, twice a day for 28 days.	Probing	Improvement of GI and PI.	[127], 2015
Clinical pilot study	Japan	Patients with advanced periodontitis, aged 41–64 years, PPD ≥ 5 mm.	6	Placed strips with 5% green tea extract into the periodontal pocket, once a week for 8 weeks.	GCF test, probing, and peptidase activity test.	Improvement of PD and the proportion of BPR and lower peptidase activity.	[113], 2002
Animal study	South Korea	Sprague-Dawley rats with periodontitis induced by ligature tying.	48	Administrated with 200 mg·kg^−1^ of EGCG via oral gavage daily for 1, 2, or 4 weeks.	Histologic analysis and IHC	Reduced CEJ-ABC distance and destruction of long junctional epithelium and collagen, and downregulated gene expression of IL-6 and TNF.	[118], 2013
Animal study	China	7–8-week-old C57BL/6 mice with periodontitis induced by ligature tying.	33	Sterile cotton swabs containing 0.312–1.25 mg·mL^−1^ of EGCG inserted into mouths in 2 min for 7 days.	Histologic analysis, micro-CT, ELISA, and cultured GCF	Reduced CEJ-ABC distance, and decreased IL-6 in the serum and abundance of microorganisms.	[109], 2023
Animal study	China	8-week BALB/c mice fed with *Porphyromonas gingivalis* FDC381 per 2 days for 40 days.	24	Replaced distilled water with 0.02% EGCG solution as feeding.	Histological analysis, micro-CT, IHC, ELISA, and qPCR	Reduced CEJ-ABC distance, and downregulated expression of IL-1β, IL-6, IL-17, IL-23, and TNF-α.	[119], 2015

RCT, randomized controlled trial; EGCG, epigallocatechin gallate; CP, chronic periodontitis; PPD, probing pocket depth; CAL, clinical attachment level; GI, gingival index; PI, plaque index; BI, bleeding index; *w*/*w*, weight to weight ratio; rCAL, relative clinical attachment level; GCF, gingival crevicular fluid; BPR, black-pigmented Gram-negative anaerobic rods; IHC, immunohistochemistry; CEJ-ABC, cementoenamel junction to the alveolar bone crest; IL, interleukin; TNF, tumor necrosis factor; ELISA, enzyme-linked immunosorbent assay; qPCR, quantitative polymerase chain reaction.

**Table 6:** The effects of EGCG on oral cancer cells in in vitro studies.

Cells	Intervention	Relevant Findings	Reference, Year
hGF-1, SCC-1, and SCC-9	100 μM of EGCG	Induced intracellular ROS in SCCs but not in hGFs, inhibited gene expression of SIRT3 in SCC-25 via ERRα, and increased activity of SIRT3 in hGFs.	[132], 2015
dominant-negative RhoA N19 SCC and constitutively active RhoA Q63E SCC	50 μM of EGCG	Inhibited invasion of SCCs and invadopodia formation without affecting viability, inhibited Src, FAK, and CTTN phosphorylation and MMPs activity, and declined RhoA activity.	[167], 2013
TSCC CAL27 and SCC-15	0–200 μM of EGCG	Inhibited proliferation, migration, and invasion, promoted apoptosis, decreased p-Akt and EMT, downregulated Hippo-TAZ signaling, and synergistic effects with simvastatin.	[168], 2018
TSCC Tca8113 and TSCCa	0–80 μM of EGCG	Inhibited the anchorage-independent growth dose-dependently and inhibited HK2 expression and glycolysis via EGFR-Akt signaling pathway to promote apoptosis.	[169], 2015
hGF-1, SCC-9, and SCC-25	0–200 μM of EGCG	Inhibited growth and cell viability, promoted apoptosis, induced extracellular ROS, induced intracellular ROS in SCCs but not in hGFs, upregulated oxidative stress genes in SCCs, but downregulated them in hGFs, and downregulated antioxidant genes in SCCs, but upregulated them in hGFs.	[170], 2014
SCC-HSC4, HSC3, HSC2, and SAS	100 μM of EGCG	Downregulated gene expression of histamine H1R and SLC22A3, and increased gene expression of HDC and histamine production.	[171], 2022
SCC-H400 and SCC-H357	10 and 20 μg·mL^−1^ of EGCG	Inhibited cell proliferation and migration time-dependently, and decreased phosphorylation of EGFR.	[172], 2019
SCC-25 and SAS	0–50 μM of EGCG	Inhibited cell proliferation, arrested cell cycle at the G_1_ phase via upregulating BTG2 expression, and induced phosphorylation of JNK, ERK, and p-38 in SAS.	[173], 2015
SCC CAL27, SCC-4 and SCC-9	50 μM of EGCG	Inhibited cell migration via inhibiting MMP-2 activity and gene expression.	[174], 2022
SCC-PE/CA-PJ15 and SCC H357	100 μM of EGCG, combined with 3.12–200 μM of 5-FU and radiation	Synergistically reduced cell viability and migration, arrested, and increased proportion of cells in the G_2_/M phase with the addition of EGCG.	[175], 2019
HNSCC OECM1, SAS, HSC3, and FaDu	0.1–100 μM of EGCG, combined with ALA-PDT	Inhibited ABCG2 expression and modulated PpIX accumulation and ALA-PDT efficiency.	[176], 2020
SCC CAL27	1–300 μM of EGCG	Inhibited cell migration, cell invasion, and the gene expression and secretion of MMPs enhanced by AG-9.	[177], 2020
SCC-3	0–100 μM of EGCG	Inhibited cell proliferation, arrested cell cycle at the G_1_ checkpoint, and promoted apoptosis via increasing activation of caspase-3 and -7.	[178], 2019

hGF, human gingival fibroblast; SCC, human oral squamous cell carcinoma cell; M, mol·L^−1^; EGCG, epigallocatechin gallate; ROS, reactive oxygen species; SIRT3, sirtuin 3; ERRα, estrogen-related receptor α; RhoA, ras homolog gene family, member A; Src, sarcoma gene; FAK, focal adhesion kinase; CTTN, cortactin; MMP, matrix metalloproteinase; TSCC, tongue squamous cell carcinoma cell; p-Akt, phosphorylated protein kinase B; EMT, epithelial mesenchymal transition; TAZ, transcriptional coactivator with PDZ-binding motif; HK2, hexokinase2; EGFR, epidermal growth factor receptor; SLC22A3, solute carrier 22A3; HDC, L-histidine decarboxylase; BTG2, B-cell translocation gene 2; JNK, c-Jun N-terminal kinase; ERK, extracellular regulated protein kinase; 5-FU, 5-fluorouracil; ALA-PDT, 5-aminolevulinic acid photodynamic therapy; ABCG2, ATP-binding cassette G2; PpIX, protoporphyrin IX; HNSCC, head and neck squamous cell carcinoma cell; AG-9, an elastin nonapeptide of consensus sequence xGxPGxGxG, AGVPGLGVG.

**Table 7:** The effects of green tea extract or EGCG on oral cancer in in vivo studies.

Study Type	Country	Subject	Sample Size	Intervention	Analysis	Relevant Findings	Reference, Year
Animal study	South Korea	male BALB/c athymic nude mice aged 6 weeks, with tongue tumors established	10	20 mg·kg^−1^ of EGCG, intraperitoneally injected once per 2 days for 4 weeks	IHC	Reduced tumor volume and inhibited Src, FAK, and CTTN, and reduced regional infiltration via inhibition of Src substrate phosphorylation and MMPs activities.	[167], 2013
Animal study	Japan	female BALB/c nude mice aged 5 weeks, with SCC-3 implanted into mice back	20	75 mg·kg^−1^ of EGCG, intraperitoneally injected twice a week for 4 weeks	IHC, and TUNEL staining	Reduced tumor volume, inhibited cell proliferation, and promoted apoptosis.	[178],2019
Animal study	China	BALB/c nude mice aged 5 weeks, with SCC KBV200 implanted into mice back	20	25 mg·kg^−1^ of EGCG, intraperitoneally injected once per 2 days for 2 months	Histology analysis and IHC	Inhibited the onset and growth of tumors, slowed down slightly the body weight loss, and prolonged the overall survival time.	[181], 2022
Animal study	China	BALB/c nude mice aged 5 weeks, with SCC KBV200 implanted into the right axilla	12	10, 20, and 40 mg·kg^−1^ of EGCG, combined with 0.46 mg·kg^−1^ of VCR, intraperitoneally injected once a day for 13 days	Histology analysis, IHC, ELISA, and sqPCR	Inhibited growth of medium and small vessels, sensitized multidrug-resistant tumors to VCR, reduced weight loss via reducing toxicity of VCR, and reduced gene expression and secretion of VEGF.	[182], 2020
Animal study	USA	Female athymic nude mice aged 4–6 weeks, with HNSCC Tu212 implanted into the right flank	20	125 mg·kg^−1^ of EGCG, combined with 30 mg·kg^−1^ resveratrol, orally gavaged 5 times a week for 4 weeks	IHC, and TUNEL staining	Synergistically inhibited tumor growth and induced apoptosis, synergistically inhibited Akt-mTOR signaling, and decreased Mcl-1 and survivins.	[180], 2021
Phase I b clinical study	USA	Patients with documented histology of premalignant lesions of the oral cavity or larynx	21	200 mg GPP orally administered 3 times a day, and erlotinib orally administered daily with dose escalation from 50 mg (level 1) to 75 mg (level 2), and to 100 mg (level 3) for 6 months	Biopsy and biomarker studies	The recommended dose of GPP was 600 mg per day; pathologic complete response (47%) and pathologic partial response (18%); the 5-year CFS and OS were 66.3% and 93%; and decreased expression of pERK.	[188], 2020
Double-blind RCT	India	Patients with histologically confirmed and bidimensionally assessable OPMDs	60	400 mg green tea extract and 475 mg curcumin, topical or systemic administration, twice a day for 3 months	Biopsy and biomarker studies	Higher clinical response rate with combination group; insignificantly improved histological grades; significantly downregulated gene expressions of p53, Ki67, and cyclin D1	[183], 2020
Double-blind RCT	China	Patients aged over 20 years old, newly diagnosed with oral cancer, and treated with oral surgery within one month prior	63	100 mL infusion containing 5% green tea powder for rinsing 1 min after brushing tooth, twice a day for 6 months	oral health status evaluated according to the Oral Assessment Guide	Significant improved oral health status after 4 months	[189], 2021
Non-randomized clinical trial	China	Patients with pathologically confirmed head and neck cancer	20	15 mL mouthwash containing 440–2200 μM of EGCG for rinsing 5 min, 3 times a day until 2 weeks after the end of radiotherapy	PST, OMAS, and NRS	The recommended concentration of EGCG was 1760 μM; reduced radiation-induced oral mucosal injury; improved patient satisfaction	[150], 2020

RCT, randomized controlled trial; EGCG, epigallocatechin gallate; IHC, immunohistochemistry; Src, sarcoma gene; FAK, focal adhesion kinase; CTTN, cortactin; MMP, matrix metalloproteinase; SCC, human oral squamous cell carcinoma cell; TUNEL, terminal deoxynucleotidyltransferase-mediated dUTP-biotin nick end labeling; VCR, vincristine sulfate; ELISA, enzyme-linked immunosorbent Assay; sqPCR, semiquantitative polymerase chain reaction; VEGF, vascular endothelial growth factor; HNSCC, head and neck squamous cell carcinoma cell; Akt, protein kinase B; mTOR, mammalian target of rapamycin; CFS, cancer-free survival; OS, overall survival; GPP, green tea polyphenol; pERK, phosphorylated extracellular regulated protein kinase; OPMD, oral potentially malignant disorders; M, mol·L^−1^; PST, WHO, patient satisfaction tool; OMAS, oral mucositis assessment scale; NRS, numerical rating scale.

## Data Availability

No new data were created or analyzed in this study.
